# Case Report: Prenatal Whole-Exome Sequencing Identified a Novel Nonsense Mutation of the *KCNH2* Gene in a Fetus With Familial 2q14.2 Duplication

**DOI:** 10.3389/fgene.2022.924573

**Published:** 2022-07-05

**Authors:** Jianlong Zhuang, Chunnuan Chen, Yuanbai Wang, Shuhong Zeng, Yu’e Chen, Yuying Jiang, Yingjun Xie, Gaoxiong Wang

**Affiliations:** ^1^ Prenatal Diagnosis Center, Quanzhou Women’s and Children’s Hospital, Quanzhou, China; ^2^ Department of Neurology, The Second Affiliated Hospital of Fujian Medical University, Quanzhou, China; ^3^ Ultrasonography, Quanzhou Women’s and Children’s Hospital, Quanzhou, China; ^4^ Department of Obstetrics and Gynecology, Guangdong Provincial Key Laboratory of Major Obstetric Diseases, The Third Affiliated Hospital of Guangzhou Medical University, Guangzhou, China; ^5^ Key Laboratory of Reproduction and Genetics of Guangdong Higher Education Institutes, The Third Affiliated Hospital of Guangzhou Medical University, Guangzhou, China; ^6^ Quanzhou Women’s and Children’s Hospital, Quanzhou, China

**Keywords:** chromosomal array analysis, *KCNH2*, tetralogy of fallot, LQTS, whole-exome sequencing

## Abstract

**Background:** Pathogenic mutations in the *KCNH2* gene were associated with long QT syndrome 2 (LQT2), which typically manifest in a prolonged QT interval and may lead to recurrent syncopes, seizure, or sudden death. Limited reports indicated that the *KCNH2* mutations would result in LQT2 combined with tetralogy of fallot. Our goal was to present an additional case of LQT2 combined with the tetralogy of fallot in a fetus with a novel *KCNH2* mutation.

**Case presentation:** Enrolled in this study was a 23-year-old pregnant woman from Quanzhou Fujian province, China. In her pregnancy, fetal ultrasound anomalies were identified, including tetralogy of fallot, coronary sinus enlargement, and persistent left superior vena cava. No chromosomal abnormality was detected by fetal karyotype analysis. However, 238.1-kb duplication in the 2q14.2 region containing the *GLI2* gene was observed in the fetus by chromosomal array analysis, which was inherited from the mother with normal clinical features and interpreted as a variant of uncertain significance (VOUS). Furthermore, whole-exome sequencing (WES) detection identified a novel nonsense c.1907C > G (p.S636*) mutation in the *KCNH2* gene in the fetus, and it was classified as a likely pathogenic variant, according to the ACMG guidelines. Parental verification analysis indicated that c.1907C > G (p.S636*) mutation was inherited from the mother.

**Conclusion:** In this study, we believe that 2q14.2 duplication may not be the reason for fetal heart defects; moreover, we described an additional case with *KCNH2* gene mutation, which may lead to LQTS and be associated with congenital heart defects. In addition, our study further confirms the application value of the WES technology in prenatal genetic etiology diagnosis of fetuses with structural anomalies and unexplained structural variants.

## Introduction

### Background

Microdeletions or microduplications in the 2q14.2 region are extremely rare variants, with approximately 10 case reports being available in the literature. The 2q14.2 microscopic deletions usually manifest phenotypic variability and incomplete penetrance; the clinical phenotypes are typically characterized as holoprosencephaly, abnormal pituitary gland formation and/or function, craniofacial dysmorphisms, branchial arch anomalies, and polydactyly (Kevelam et al., 2012; Kordaß et al., 2015; Elizabeth et al., 2020). Isolated 2q14.2 duplications are rarer with no defined specific syndrome. To the best of our knowledge, only one report was referred to 2q14.2 duplication, and it demonstrated that it may be responsible for microphthalmia/microcornea and congenital cataracts in an affected family (Schilter et al., 2013).

Congenital long QT syndrome (LQTS) is a genetic disorder that typically manifests arrhythmia of a prolonged QT interval and a risk of recurrent syncopes, seizures, or sudden death (Crotti et al., 2008; Hedley et al., 2009; Christiansen et al., 2014). A previous study indicated that LQTS showed a high incidence of approximately 1/2,500 in healthy live births (Schwartz et al., 2009). Among them, *KCNQ1*, *KCNH2* (*HERG*), and *SCN5A* are the three most frequently affected genes, which would lead to LQT1, LQT2, and LQT3, respectively. The main mutation types in LQT2 are nonsense mutation and missense mutation, with more than half of them being nonsense mutations and predict to result in haploinsufficiency through nonsense-mediated RNA decay (Ono et al., 2020). At present, no specific forms of congenital heart defects (CHD) have been reported to associate with LQT2, while several cases with *KCNH2* mutations showed coexistence of CHD (Bhuiyan et al., 2014; Ebrahim et al., 2017; Song et al., 2018).

In this study, the whole-exome sequencing (WES) technology was first employed for further prenatal diagnosis in a fetus who harbored familial 2q14.2 duplication and exhibited fetal congenital heart defects including tetralogy of fallot. In addition, our study further strengthened the application value of the WES technology in genetic etiology of fetuses with structural anomalies and unexplained copy number variants.

## Case Presentation

**FIGURE 1:**
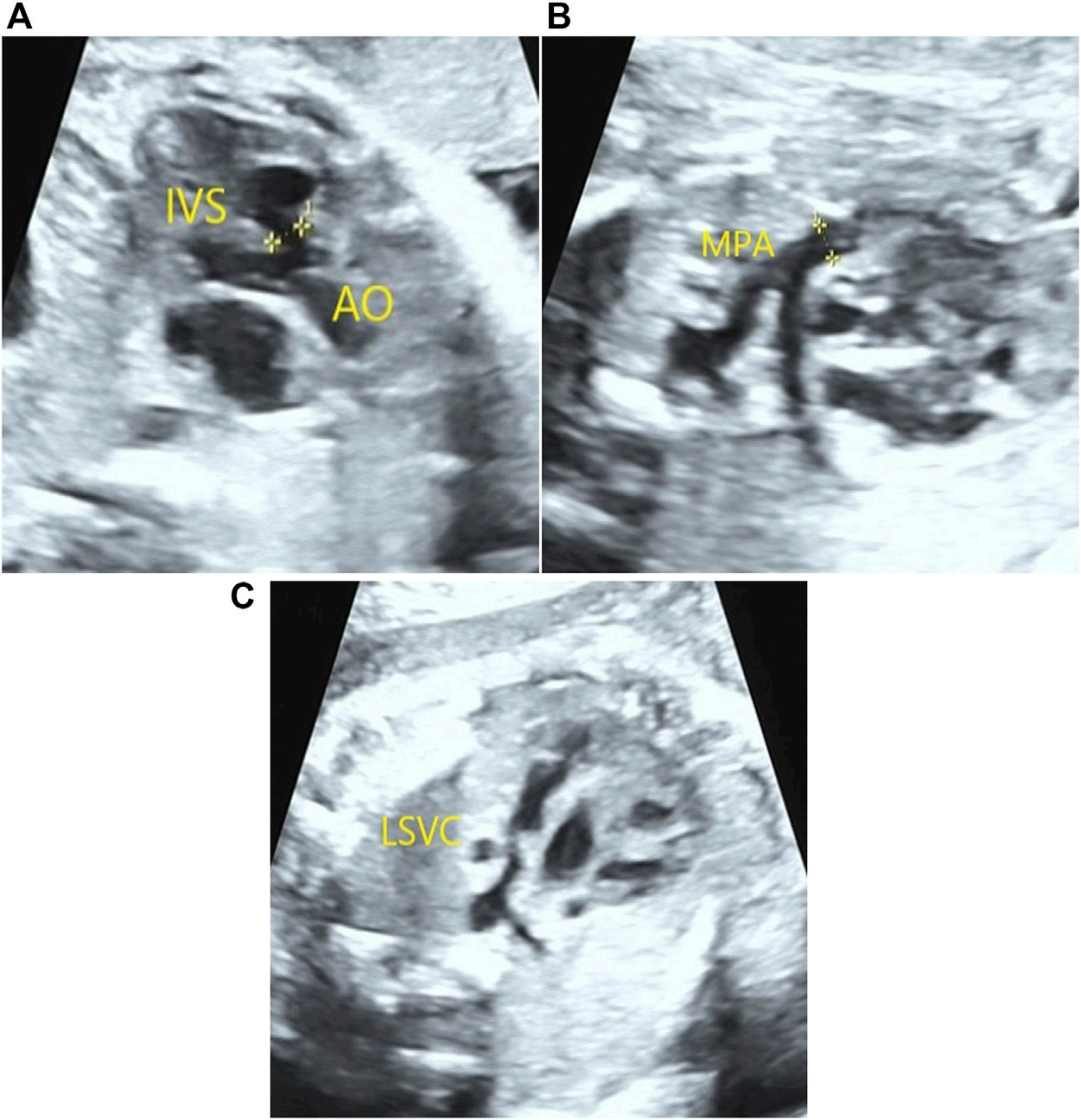
Congenital heart defects detected in the fetus by prenatal ultrasound examination. A 0.41-cm ventricular septal defect and overriding aorta **(A)** were detected in the fetus by prenatal ultrasound examination. In addition, pulmonary artery stenosis **(B)** and persistent left superior vena cava **(C)** were also observed.

A 23-year-old Chinese pregnant woman, gravida 1, para 0, from Quanzhou Fujian province, was enrolled in this study. Her husband was 29 years old, and they denied consanguinity marriage and had a family history of inherited disease. In this pregnancy, low progesterone was observed in the first trimester and was treated with didroxyprogesterone. No obvious abnormality was found by ultrasound examination during her first-trimester pregnancy, with a normal nuchal translucency (0.7 mm). In the second trimester, a low-risk screening result was detected by Down’s screening. However, at the gestational age of 23^+2^ weeks, three-level color Doppler ultrasound examination results revealed abnormal heart defects in the fetus, including tetralogy of fallot (ventricular septal defect, overriding aorta, and pulmonary artery stenosis), coronary sinus enlargement, and persistent left superior vena cava (Figure 1). Amniocentesis was performed at the gestational age of 25^+5^ weeks, after sufficient genetic consultation.

No obvious chromosomal abnormality was detected in the fetus by karyotype analysis, and her parents showed normal karyotypes as well. However, chromosomal array analysis (CMA) results demonstrated a 238.1-kb duplication in the 2q14.2 region ([GRCh37]2q14.2 (121,477,769–121,715,896)x3) in the fetus containing the *GLI2* gene (OMIM:165230). According to the ACMG guidelines, the 2q14.2 duplication was interpreted as variants of uncertain significance. Parental CMA verification demonstrated that the 2q14.2 duplication was inherited from the mother who exhibited normal clinical features.

**FIGURE 2:**
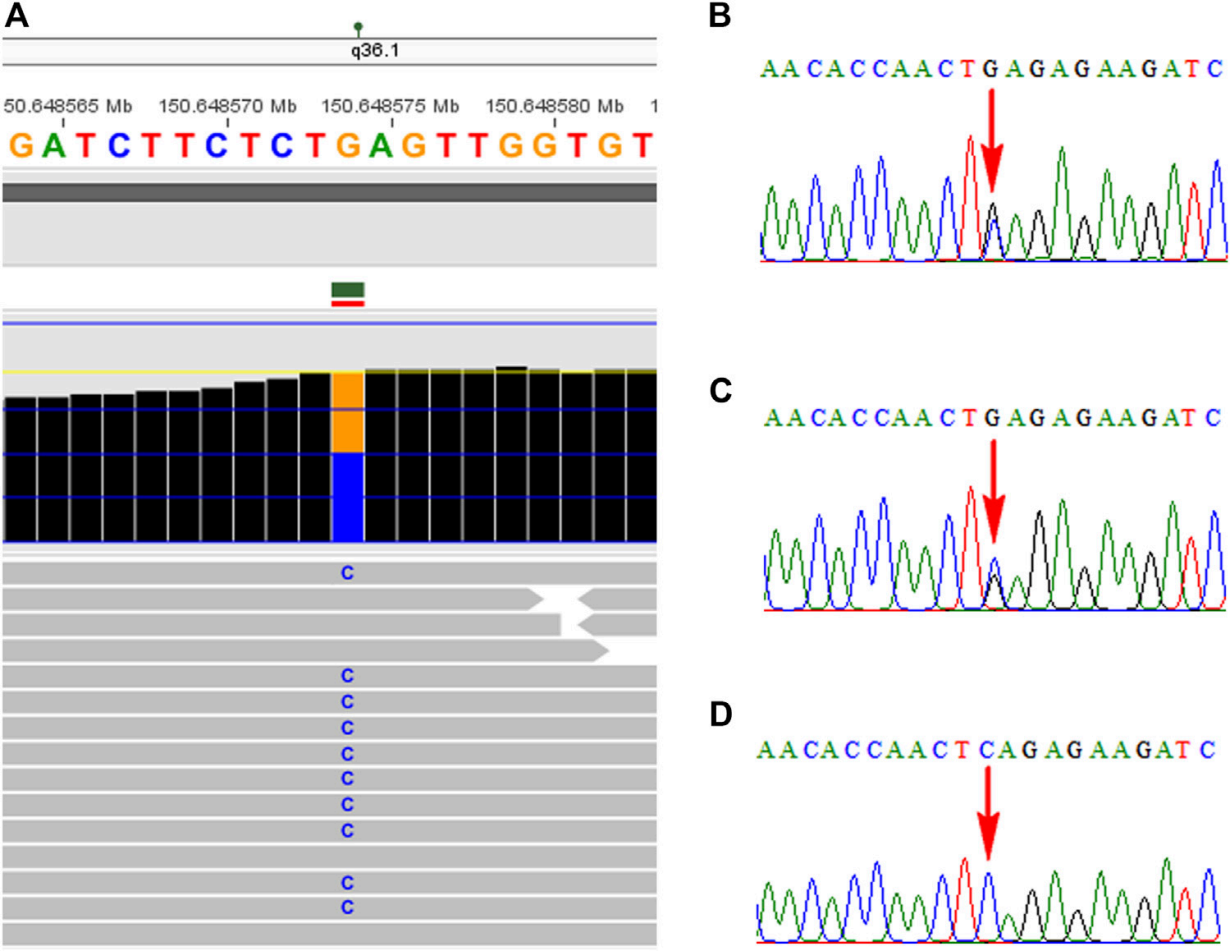
Identification of the *KCNH2* mutation in the family using the WES technology and Sanger sequencing. **(A)** Novel nonsense mutation in the *KCNH2* gene was identified in the fetus by WES. **(B)** Mutation of c.1907C > G was confirmed by Sanger sequencing. Parental Sanger sequencing revealed that her mother carried the same mutation **(C),** while the mutation was not found in her father **(D)**.

WES technology was carried out to look for additional variants in the fetus with unexplained structural variants of 2q14.2 duplication. A novel nonsense mutation of c.1907C > G (p.S636*) in the *KCNH2* gene was identified in the fetus by WES technology, which was inherited from the mother and further confirmed by Sanger sequencing (Figure 2). No frequency was found in the databases of gnomAD, 1,000 genomes, dbSNP, and ExAC. In addition, several computer-aided analysis software applications predicted that this variation may affect the protein structure/function (MetaSVM_score: 0.717; GERP++_RS: 5.14; dbscSNV_ADA: 0; dbscSNV_RF: 0). According to the ACMG Guidelines (Richards et al., 2015), the nonsense mutation was interpreted as a likely pathogenic variant (PVS1, PM2_Supporting). At present, no obvious cardiac abnormality was observed in the pregnant woman by electrocardiogram and echocardiography. Finally, the family chose to terminate her pregnancy at a gestational age of 30 weeks.

## Discussion and Conclusion

With the advanced application of the CMA technology, an increasing rate of variants of uncertain significance (VOUS) and variants with phenotypical diversity may be followed, which will result in a big challenge for genetic consultation. The WES technology showed a great effectiveness in genetic etiology diagnosis in patients at the single-gene level, while few studies were available to further reveal the phenotypical diversity of unexplained structure variants using WES technology. Additional mutations besides the structural variants were identified by WES detection in patients with familial 1q21.1 microduplication/microdeletion, duplication of Xp22.31, and 16p11.2 duplication who exhibited phenotypical variability (Dastan et al., 2016; Qiao et al., 2017; Qiao et al., 2019). In addition, a recent study conducted by Granata et al. (2022) identified variants in *CECR2*, *MTOR*, *RICTOR,* and *LRRK2* genes by WES detection in the affected patients who also had a 16p13.11 microdeletion and proposed that WES technology could be used as a fundamental tool to identify additional mutations in patients with a predisposing variant. In this study, the WES technology was employed to further investigate the genetic etiology in a fetus with familial 2q14.2 duplication who had more severe phenotypes.

In the present study, a 238.1-kb duplication in the 2q14.2 region was identified in the fetus, which was inherited from her mother with a normal phenotype. As shown in the DECIPHER database and listed in Table 1, all small fragments of 2q14.2 duplication were interpreted as VOUS without specific syndrome. In addition, as elicited in the ClinGen database, the dosage sensitivity of *GLI2* gene indicated sufficient evidence of haploinsufficiency (3) but without triplosensitivity evidence (0). In addition, a patient harbored both 2q14.1q14.2 duplication and 2q37.3 deletion and exhibited autism, while the authors believed that the clinical phenotype may ascribe to 2q37.3 deletion (Devillard et al., 2010). In this study, we believe that the 2q14.2 duplication may not contribute to the ultrasonic abnormalities in the fetus. However, a previous study indicated that 2q14.2 duplication was co-segregated with microphthalmia/microcornea and congenital cataracts in an affected family and suggested that the 2q14.2 duplication may be the reason for the clinical features (Schilter et al., 2013). Nevertheless, no obvious ocular abnormalities were observed in our study, and the congenital heart defects including tetralogy of fallot in the fetus were hard to be explained by 2q14.2 duplication.

**TABLE 1:** Cases with less than 1.0-Mb duplications in the 2q14.2 region are presented in the DECIPHER database.

DECIPHER Patients	Sex	Location (GRCh38)	Size (kb)	Inheritance	Pathogenicity	Phenotype
404183	46,XX	2:120793535–120842777	49.24	Unknown	Uncertain	NM
300369	NM	2:120842718–121028767	186.05	Maternally	Uncertain	Cognitive impairment and generalized hypotonia
383370	46,XX	2:120102148–120751795	649.65	Unknown	Uncertain	Abnormal lateral ventricle morphology, abnormal third ventricle morphology, autism, delayed speech and language development, scoliosis, and thick corpus callosum
384452	46,XY	2:120113202–120975414	862.21	Unknown	Uncertain	Behavioral abnormality
Our case	46,XX	2:120720193–120958320	238.1	Maternally	Uncertain	Tetralogy of fallot, coronary sinus enlargement, and persistent left superior vena cava

NM: not mentioned.

Further WES detection revealed a novel nonsense mutation of c.1907C > G (p.S636*) in the *KCNH2* gene in the fetus, which was interpreted as a likely pathogenic variant. In addition, no pathogenic or uncertain variants in the known genes that referred to CHD features were identified in the fetus. *KCNH2* encodes the pore-forming subunit of a rapidly activating delayed rectifier potassium channel; loss-of-function mutations in the *KCHN2* gene would lead to LQT2 (Gianulis and Trudeau, 2011). As delineated by previous studies, several patients diagnosed with long QT syndrome are also accompanied by congenital heart defects (Massin et al., 2010; Hsiao et al., 2007; Wu et al., 1999). The study conducted by Murugan et al. (2005) presented a family with LQTS and coexisting persistent patency of the arterial duct, and they proposed that it may not be a coincidence, and hypothesis about a possible genetic mechanism may exist. In addition, a new form of LQTS was indicated in three patients who also had LQTS and associated with structural heart disease and syndactyly (Marks et al., 1995). Moreover, a previous study (Ebrahim et al., 2017) presented 11 patients who harbored single-gene mutations that resulted in long QT syndrome, combined with congenital heart defects. Among them, four patients carried *KCNQ1* gene mutation, and one patient had a *KCNQ1* mutation associated with *KCNH2* mutation. Most of them (six cases) had *KCNH2* mutations, of which two cases had tetralogy of fallot (TOF) (Ebrahim et al., 2017). In addition, patients with *KCNH2* mutations who had LQTS combined with tetralogy of fallot (TOF) are summarized in Table 2. In the present study, the fetus had a novel *KCNH2* mutation and manifested congenital heart defects including TOF, which further enhanced the genotype–phenotype correlation.

**TABLE 2:** *KCNH2* mutations in patients who had LQTS combined with tetralogy of fallot in the literature.

	(Ebrahim et al., 2017)		(Bhuiyan et al., 2014)		(Chiu et al., 2012)		Our case
	Patient 3	Patient 11	Sister 1	Sister 2	Case 1	Case 2	
Sex/Age	NA	NA	F/13	F/11	M/NA	F/NA	F/Fetus
Genes	KCNH2	KCNH2	KCNH2	KCNH2	KCNH2/SCN5A	KCNH2/SCN5A	KCNH2
Mutations	G1036_	G572S	p.[(V172M); (R293C)]	p.[(V172M); (R293C)]	p.M645R/p.R1193Q	p.M645R/p.R1193Q	p.S636*
	L1042del						
CHD	TOF	TOF	TOF	TOF	TOF	TOF	TOF
QTc (ms)	635	510	450	NA	581	641	NA
Inheritance	NA	NA	Maternal	Maternal	NA	NA	Maternal

NA: not available; F: female; M: male; CHD: congenital heart defects; TOF: tetralogy of fallot.

In addition, the *KCNH2* mutation in the fetus was inherited from her mother, who had no obvious cardiac phenotype. Similar to a previous study (Bhuiyan et al., 2014), two sisters in a close relative married family were found to carry the *KCNH2* mutation, which was also inherited from their normal mother, which suggests incomplete penetrance of *KCNH2*, although it cannot be ruled out that the phenotype may appear in her mother in the future. Therefore, our study indicated that the additional variant in the *KCNH2* gene identified in the fetus may be responsible for fetal ultrasound anomalies, rather than the 2q14.2 duplication. The fetus is likely to harbor a LQT2; unfortunately, a fetal electrocardiogram *in utero* was not available in this study, and whether the fetus has a prolonged QT interval is unknown. Moreover, the nonsense mutation of the *KCNH2* gene identified in the fetus is classified as a secondary finding, according to the ACMG secondary finding v3.0 list; no sufficient evidence was available to reveal the causal relationship between *KCNH2* mutations and CHD so far. Thus, more work needs to be conducted to clarify the existence of a genetic mechanism or if it was just a coincidence between *KCNH2* mutations and CHD.

In conclusion, our study described an additional case of LQT2 combined with tetralogy of fallot in a fetus with a *KCNH2* mutation. In addition, our study enriched the mutation spectrum of the *KCNH2* gene and further indicated the application value of WES in prenatal diagnosis in fetuses with familial uncertain copy number variants.

## Data Availability

The datasets for this article are not publicly available due to concerns regarding participant/patient anonymity. Requests to access the datasets should be directed to the corresponding author.
